# Global centers of a family of cubic systems

**DOI:** 10.1007/s00010-024-01051-7

**Published:** 2024-04-05

**Authors:** Raul Felipe Appis, Jaume Llibre

**Affiliations:** 1 Departamento de Matemática, Universidade Federal de São Carlos, São Paulo, São Carlos 13565-905 Brazil; 2 Departament de Matemàtiques, Universitat Autònoma de Barcelona, 08193 Bellaterra, Barcelona, Catalonia Spain

**Keywords:** Global center, Center, Cubic system, Primary 34A34, Secondary 34C25, 37C37, 14R15

## Abstract

Consider the family of polynomial differential systems of degree 3, or simply cubic systems

$$ x' = y, \quad y' = -x + a_1 x^2 + a_2 xy + a_3 y^2 + a_4 x^3 + a_5 x^2 y + a_6 xy^2 + a_7 y^3, $$

in the plane $\mathbb {R}^2$. An equilibrium point $(x_0,y_0)$ of a planar differential system is a *center* if there is a neighborhood $\mathcal {U}$ of $(x_0,y_0)$ such that $\mathcal {U} \backslash \{(x_0,y_0)\}$ is filled with periodic orbits. When $\mathbb {R}^2\setminus \{(x_0,y_0)\}$ is filled with periodic orbits, then the center is a *global center*. For this family of cubic systems Lloyd and Pearson characterized in Lloyd and Pearson (Comput Math Appl 60:2797–2805, 2010) when the origin of coordinates is a center. We classify which of these centers are global centers.

## Introduction and statement of the main result

Let $P,Q: \mathbb {R}^2 \longrightarrow \mathbb {R}$ be polynomials and consider the differential system

1 $$\begin{aligned} x' = P(x,y), \qquad y' = Q(x,y). \end{aligned}$$

Denote by $X(x,y)=(P(x,y),Q(x,y))$ the vector field associated to the differential system (1). The *degree* *d* of system (1) is the maximum of the degrees of the polynomials *P* and *Q*. Here the apostrophe denotes derivative with respect to the time *t*. A point $(x_0,y_0)$ is an *equilibrium point* of system (1) if $X(x_0,y_0)=(0,0)$.

An equilibrium point $(x_0,y_0)$ of system (1) is a *center* if there is a simply connected open neighborhood *W* of $(x_0,y_0)$ such that $(x_0,y_0)$ is the only equilibrium point in *W* and all the trajectories contained in $W \backslash \{(x_0,y_0)\}$ are periodic. The largest simply connected open neighborhood $\mathcal {P}$ of $(x_0,y_0)$ such that $\mathcal {P} \backslash \{(x_0,y_0)\}$ is filled of periodic trajectories is called the *period annulus*. When $\mathcal {P}=\mathbb {R}^2$ the point $(x_0,y_0)$ is a *global center*.

Dulac [5] and Poincaré [13] were the first in studying the centers of the differential systems in the plane. While Conti [4] was the first in studying the global centers.

To classify the centers of polynomial differential systems as well as to determine the necessary and sufficient conditions to know whether a center is global are in general difficult problems.

Kapteyn [8] and Bautin [2] classified the centers of the polynomial differential systems of degree 2, the quadratic centers. For degrees higher than 2 the classification of all centers remain unsolved.

Galleotti and Villarini [7] proved that polynomial differential systems of even degree do not admit global centers, a shorter proof was given in [10].

The classification of the global centers for homogeneous polynomial differential systems is well known, see for instance [3]. The classification of the global centers of quasi-homogeneous polynomial differential systems is also well studied, see [9].

In this paper we classify the global centers of the following family of cubic polynomial differential systems

2 $$\begin{aligned} x' = y,\qquad y' = -x + a_1 x^2 + a_2 xy + a_3 y^2 + a_4 x^3 + a_5 x^2 y + a_6 xy^2 + a_7 y^3. \end{aligned}$$

Lloyd and Pearson in [12] classified when the origin of coordinates of systems (2) is a center. This result is stated as follows.

### Theorem 1

The origin of system (2) is a center if and only if one of the following five conditions holds:

(i) $$a_2=a_5=a_7=0$$;
(ii) $$a_1=a_3=a_5=a_7=0$$;
(iii) $$a_4=a_3(a_1+a_3)$$, $$a_5=-a_2(a_1+a_3)$$ and $$(a_1+2a_3)a_6+a^2_3(a_1+a_3)=a_7=0$$;
(iv) $$a_5+3a_7+a_2(a_1+a_3)=0$$, $$9a_6a^2_2 + 2a^4_2 + 27 a_7 \mu + 9 \mu ^2=0$$, $$a_4 a^2_2 + a_5 \mu =0$$, $$(3a_7 \mu +\mu ^2 + a_6 a^2_2) a_5 - 3a_7 \mu ^2-a_6a^2_2 \mu =0$$ where $$\mu =3a_7 +a_2 a_3$$;
(v) $$a_5 + 3a_7 +a_2(a_1+a_3)=0$$, $$18 a_4 a_5 - 27a_4 a_7 + 9a_5a^2_1 +9a_5 a_6 +2a_5 a^2_2=0$$, $$27 a_4 a_1 + 4a_5 a_2 + 9a^3_1+2a_1 a^2_2=0$$, $$18a^2_4 + 9a_4 a^2_1 + 2a_4 a^2_2 +2a^2_5=0$$, $$18 a_4 a_2 + 9a_5 a_1 + 9a_5 a_3 +9a^2_1 a_2-27a_1 a_7+9a_6a_2 + 2a^3_2=0$$.

The next two results help for classifying the global centers of the cubic polynomial differential systems (2).

### Proposition 2

If the origin of a differential system (2) is a global center, then $a_7=0$.

### Proposition 3

A differential system (2) has the unique equilibrium point (0, 0) if and only if either $a_1=a_4=0$, or $a^2_1 + 4 a_4<0$.

In the following result we classify the global centers of system (2). In what follows from Proposition 2 we assume that $a_7=0$, and from Proposition 3 that $a_4\le 0$.

### Theorem 4

Assume that the unique equilibrium of the differential system (2) is the (0, 0). Under the condition (i) of Theorem 1 the cubic polynomial differential system (2) has a global center if and only if one the following two conditions holds:

- $$a_6<0$$;
- $$a_3=a_6=0$$ , $$a_4<0$$.

Under the condition (ii) of Theorem 1, the cubic polynomial differential system (2) has a global center if and only if one the following three conditions holds:

(c) $$a_4=0$$ and $$a_2^2+4a_6<0$$;
(d) $$a_4<0$$ and $$a_6<0$$;
(e) $$a_6=0$$ and $$a^2_2 + 8 a_4 < 0$$.

Under the conditions (iii), (iv) and (v) of Theorem 1, the classification of the global center of system (2) is reduced to conditions (i) and (ii).

In Sect. 2 we present some tools that we need for proving Theorem 4. In Sect. 3 we initially prove Propositions 2 and 3 and after we prove Theorem 4.

## Preliminary results

### The Poincaré compactification

To determine conditions in order that a center of a polynomial differential system in $\mathbb {R}^2$ be global, we need to study the behavior of the flow at infinity, so we recall the Poincaré compactification of a polynomial differential system (1), essential for the study of the dynamics in a neigborhood of the infinity of the polynomial differential systems.

Let $\mathbb {R}^2 \equiv \{(x_1,x_2,1); x_1,x_2 \in \mathbb {R}\}$ and the sets $H_{+} = \{(x_1,x_2,x_3) \in \mathbb {S}^2; x_3>0\}$, $H_{-} = \{(x_1,x_2,x_3) \in \mathbb {S}^2; x_3<0\}$ and $\mathbb {S}^{1} \equiv \{(x_1,x_2,x_3) \in \mathbb {S}^2; x_3=0\}$, where $\mathbb {S}^2 = \{(x_1,x_2,x_3) \in \mathbb {R}^3; x^2_1+x^2_2+x^2_3=1\}$. In order to study a vector field over $\mathbb {S}^2$ we consider six local charts that cover the whole sphere $\mathbb {S}^2$. So, for $i=1,2,3$, let

$$ U_{i}= \{(x_1,x_2,x_3) \in \mathbb {S}^2; x_i > 0\} \text{ and } V_{i}= \{(x_1,x_2,x_3) \in \mathbb {S}^2; x_i < 0\}. $$

Consider the diffeomorphisms $\varphi _{i}: U_{i} \longrightarrow \mathbb {R}^2$ and $\psi _{i}: V_{i} \longrightarrow \mathbb {R}^2$ given by

$$ \varphi _{i}(x_1,x_2,x_3)= \psi _{i}(x_1,x_2,x_3)= \left( \dfrac{x_j}{x_i},\dfrac{x_k}{x_i} \right) $$

with $j,k \ne i$ and $j<k$. The sets $(U_i,\varphi _i)$ and $(V_i, \psi _i)$ are called the *local charts* over $\mathbb {S}^2$.

Let $f^{\pm }: \mathbb {R}^2 \longrightarrow H_{\pm }$ be the central projections from $\mathbb {R}^2$ to $\mathbb {S}^2$ given by

$$ f^{\pm }(x_1,x_2)= \pm \left( \dfrac{x_1}{\Delta (x_1,x_2)},\dfrac{x_2}{\Delta (x_1,x_2)},\dfrac{1}{\Delta (x_1,x_2)} \right) $$

where $\Delta (x_1,x_2) = \sqrt{x^2_1+x^2_2+1}$. In other words $f^{\pm }(x_1,x_2)$ is the intersection of the straight line through the points $(0,0,0),(x_1,x_2,1) \in \mathbb {R}^3$ with $H_{\pm }$. Note that $f^{+} = \varphi _{3}^{-1}$ and $f^{-} = \psi _{3}^{-1}$. Moreover, the maps $f^{\pm }$ induces over $H_{\pm }$ vector fields analytically conjugate to system (1). Indeed, $f^{+}$ induces on $H_{+}=U_3$ the vector field $X_{1}(y) = D f^{+} (\varphi _{3}(y)) X(\varphi _{3}(y))$, and $f^{-}$ induces on $H_{-}=V_3$ the vector field $X_{2}(y) = D f^{-} (\psi _{3}(y)) X(\psi _{3}(y))$. Thus we obtain a vector field on $\mathbb {S}^2 \backslash S^{1}$ that admits an analytic extension *p*(*X*) on $\mathbb {S}^2$, see for more details [6, chapter 5]. The vector field *p*(*X*) is called the *Poincaré compactification*.

Denote $(u,v)=\varphi _{i}(x_1,x_2,x_3)= \psi _{i}(x_1,x_2,x_3)$. The expression of *p*(*X*) in the chart $U_1$ is

$$ u' = v^d \left[ Q \left( \dfrac{1}{v},\dfrac{u}{v} \right) - u P \left( \dfrac{1}{v},\dfrac{u}{v} \right) \right] , v' = -v^{d+1} P \left( \dfrac{1}{v},\dfrac{u}{v} \right) . $$

The expression of *p*(*X*) in $U_2$ is

$$ u' = v^d \left[ P \left( \dfrac{u}{v},\dfrac{1}{v} \right) - u Q \left( \dfrac{u}{v},\dfrac{1}{v} \right) \right] , v' = -v^{d+1} Q \left( \dfrac{u}{v},\dfrac{1}{v} \right) . $$

The expression of *p*(*X*) in $U_3$ is

$$ u' = P(u,v), v' =Q(u,v). $$

For $i=1,2,3$ the expression of *p*(*X*) in the chart $V_i$ differs of the expression in $U_i$ only by the factor $(-1)^{d-1}$.

Note that we can identify the infinity of $\mathbb {R}^2$ with the set $\mathbb {S}^{1}$. Two points for each direction in $\mathbb {R}^2$ provide two antipodal points of $\mathbb {S}^{1}$. An equilibrium point of *p*(*X*) on $\mathbb {S}^{1}$ is called *infinite equilibrium point* and an equilibrium point on $\mathbb {S}^2 \backslash \mathbb {S}^{1}$ is called a *finite equilibrium point*. Observe that the infinite equilibrium points are in correspondence with the points (*u*, 0) on the charts $U_1,V_1,U_2$ and $V_2$. Thus, if $(x_1,x_2,0) \in \mathbb {S}^{1}$ is an infinite equilibrium point, then your antipode $(-x_1,-x_2,0)$ is also a infinite equilibrium point.

Denote by $P_{i}$ and $Q_{i}$ the homogeneous parts of degree *i* of the polynomials *P* and *Q*, respectively. Consider the polynomials

3 $$\begin{aligned} F(s)= Q_{d}(1,s)-sP_{d}(1,s) \text{ and } G(s)= P_{d}(s,1)-sQ_{d}(s,1). \end{aligned}$$

So a point $(s,0) \in \mathbb {S}^{1} \cap (U_1 \cup V_1)$ is an infinite equilibrium point if and only if $F(s)=0$. Analogously $(s,0) \in \mathbb {S}^{1} \cap (U_2 \cup V_2)$ is an infinite equilibrium point if and only if $G(s)=0$. Note that, if $(s,0) \in U_1 \cup V_1$, then

4 $$\begin{aligned} D p(X)(s,0)= \begin{bmatrix} F'(s) &{} Q_{d-1}(1,s)-sP_{d-1}(1,s) \\ 0 &{} -P_{d}(1,s) \end{bmatrix}, \end{aligned}$$

and if $(s,0) \in U_2 \cup V_2$, then

5 $$\begin{aligned} D p(X)(s,0)= \begin{bmatrix} G'(s) &{} P_{d-1}(s,1)-sQ_{d-1}(s,1) \\ 0 &{} -Q_{d}(s,1) \end{bmatrix}. \end{aligned}$$

### The vertical homogeneous blow-up

Let $(x_0,y_0)$ be an equilibrium point of system (1). Denote by $\lambda _1$ and $\lambda _2$ the eigenvalues of the Jacobian matrix $D X(x_0,y_0)$. It is said that

- $(x_0,y_0)$ is *hyperbolic* if $\lambda _1$ and $\lambda _2$ have no zero real part;
- $(x_0,y_0)$ is *semi-hyperbolic* if $\lambda _1 \lambda _2 =0$ and $\lambda ^2_1+\lambda ^2_2 \ne 0$;
- $(x_0,y_0)$ is *nilpotent* if $\lambda _1=\lambda _2=0$ and the matrix $D X(x_0,y_0)$ is not the zero matrix;
- $(x_0,y_0)$ is *linearly zero* if the matrix $D X(x_0,y_0)$ is the zero matrix.

The hyperbolic and semi-hyperbolic equilibrium points are also called *elementary equilibrium points*

In the following we present an important technique for determining the local phase portrait around an equilibrium point when it is neither hyperbolic, nor semi-hyperbolic. This method determine the local phase portrait of an equilibrium point using changes of variables called vertical blow-ups. The idea of a blow-up is to turn a non-elementary equilibrium point into a vertical straight line and study the phase portrait in a neighborhood of this straight line, applying a new blow-up to the equilibrium points which appear on this straight line if necessary. In general, such equilibrium points are less degenerate. For more details see [6, chapter 3].

We consider

$$ P(x,y) = P_{m}(x,y)+ \cdots , Q(x,y) = Q_{n}(x,y)+ \cdots $$

in system (1), where $P_{m}$ and $Q_{n}$ are homogeneous polynomials of degree $m \ge 1$ and $n \ge 1$ respectively, and the dots mean higher order terms in *x* and *y* of *m* in the polynomial *P* and of *n* in the polynomial *Q*. Consider the polynomial

$$ \mathcal {F}(x_1,x_2)=\left\{ \begin{array}{ll} xQ_{m}(x_1,x_2)-yP_{m}(x_1,x_2) &{} \text{ if } m=n \\ -yP_{m}(x_1,x_2) &{} \text{ if } m<n \\ xQ_{n}(x_1,x_2) &{} \text{ if } n<m \end{array} \right. . $$

The homogeneous polynomial $\mathcal {F}$ is called the *characteristic polynomial* of system (1) and the straight lines through the origin defined by the real linear factors of the polynomial $\mathcal {F}$ are called the *characteristic directions* at the origin, see for more details [1].

The *vertical blow-up* is the changes of variables $(x_1,x_2)\longrightarrow (u_1,u_2)$ where $(x_1,x_2)=(u_1,u_1 u_2)$. The new system in the variables $u_1$ and $u_2$ is given by

6 $$\begin{aligned} u_{1}' = x_{1}'=P(u_1,u_1 u_2), u_{2}' = \dfrac{x_1 x_{2}'-x_{1}' x_2}{x^{2}_1} = \dfrac{ Q(u_1, u_1 u_2)- u_2 P(u_1, u_1 u_2) }{u_1}. \end{aligned}$$

Note that the vertical blow-up is a diffeomorphism of $\mathbb {R}^2 \backslash \{(0,x_2)\}$ to $\mathbb {R}^2 \backslash \{(0,u_2)\}$ that swaps the second quadrant for the third quadrant, and vice versa.

The following result establishes relationships between the equilibrium at the origin of system (1) and the equilibrium points on the line $u_1=0$ of system (6), for more details see [1].

#### Theorem 5

Let $\varphi$ be a trajectory of the differential system (1) tending to origin when $t \longrightarrow +\infty$ (or $t \longrightarrow -\infty$) tangent to one of the two directions $\theta$ determined by $\tan \theta = w \ne \pm \infty$. Assume that $\mathcal {F} \not \equiv 0$. Then

(i) the straight line $\left( x_1, w x_1 \right)$ is a characteristic direction;
(ii) the point $(u_1,u_2)=\left( 0, w \right)$ is a equilibrium point of system (6) and
(iii) a trajectory $\varphi$ as in the hypothesis is in biunivocal correspondence with a trajectory of system (6) tending to an equilibrium point $\left( 0, w \right)$.

The next result provides necessary and sufficient conditions in order that a polynomial differential system in the plane $\mathbb {R}^2$ has a global center, for a proof see [11].

#### Proposition 6

A polynomial differential system in $\mathbb {R}^2$ without a line of equilibrium points at infinity has a global center if and only if it has a unique finite equilibrium point which is a center and all the local phase portraits of the infinite equilibrium points are formed by two hyperbolic sectors having all of them both separatrices on the infinite circle $\mathbb {S}^1$.

## Proofs

### Proof of Proposition 2

We have from (2) and (3) that

$$ G(s) = -s(a_4 s^3 + a_5 s^2 + a_6 s + a_7). $$

Thus the origin of the chart $U_2$ is always an infinite equilibrium point and $G'(0)=-a_7$. If $a_7 \ne 0$ then, from (5) and Theorem 2.15 of [6], the origin of the local chart $U_2$ is a hyperbolic node with eigenvalues $-a_7$ of multiplicity two. Therefore the origin of (2) cannot be global center because there are trajectories going or coming from the origin of the local chart $U_2$. Therefore $a_7=0$. $\square$

### Proof of Proposition 3

The equilibrium points of system (2) are of the form (*x*, 0) where *x* is a real root of the polynomial

$$ h(x)= x(-1 + a_1 x + a_4 x^2). $$

Therefore, the origin is the unique equilibrium point of system (2) if and only if the polynomial *h*(*x*) has no nonzero roots if and only if either $a_1=a_4=0$. or $a^2_1 + 4 a_4<0$. $\square$

### Proof of Theorem 4

By Proposition 2 we can assume $a_7=0$. From the result of Proposition 3 we divide the proof into two cases.

*Case* 1. $$a_1=a_4=0$$.

Suppose that statement (i) of Theorem 1 holds. Then $a_6 \ne 0$, otherwise the differential system (2) would have degree 2, and consequently cannot have a global center. System (2) in the chart $U_2$ writes

$$ u' = u^2v^2 - a_6 u^2-a_3uv+v^2, \qquad v' = v(uv^2-a_6u-a_3v). $$

Note that $u=0$ is not a characteristic direction at the origin of $U_2$. Doing the vertical blow up $(u,v) = (u_1,u_1 v_1)$ and eliminating with a rescaling of the time the common factor $u_1$ between $u_1'$ and $v_1'$ we obtain

$$\begin{aligned}{} & {} u_1' = P_1(u_1,v_1) \\{} & {} \quad = u_1(u_1^2 v_1^2 + v_1^2 - a_3 v_1 - a_6), \qquad v_1' = Q_1(u_1,v_1)=-v_1^3, \end{aligned}$$

with the Jacobian matrix

$$ D(P_1,Q_1)(0,0) = \begin{bmatrix} -a_6 &{} 0 \\ 0 &{} 0 \end{bmatrix}. $$

**Fig. 1:**
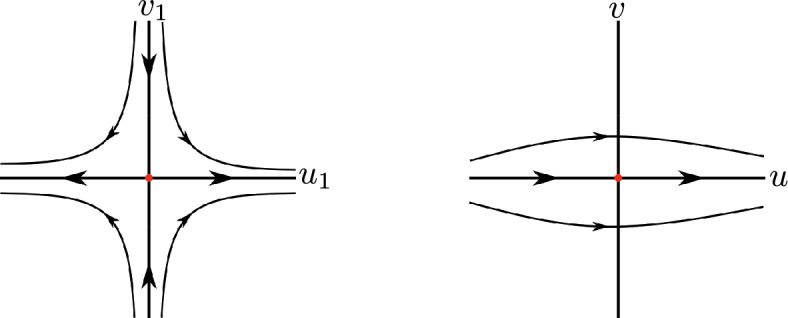
Blow up of the equilibrium point (0, 0) of the local chart $U_2$ in case 1

As $Q_1(0,v_1)= -v_1^3$ it follows, by Theorem 2.19 of [6] that, if $a_6 > 0$, then $(u_1,v_1)=(0,0)$ is a semi-hyperbolic node, and consequently system (2) cannot have a global center because there are trajectories of system (2) going or coming from the infinity. If $a_6 < 0$, then $(u_1,v_1)=(0,0)$ is a semi-hyperbolic saddle. Going back through the vertical blow up we conclude that the origin of $U_2$ is formed by two hyperbolic sectors having both separatrices at infinity, see Fig. 1.

Now system (2) in the chart $U_1$ becomes

$$ u' = -u^2v^2 + a_3 u^2v + a_6 u^2 - v^2, \qquad v' = -uv^3. $$

Since $u=0$ is not a characteristic direction at the origin of $U_1$, doing the vertical blow up $(u,v)=(u_1,u_1 v_1)$ and eliminating the common factor $u_1$ between $u_1'$ and $v_1'$ we obtain

$$ \left. \begin{aligned} u_1'&= P_1(u_1,v_1) = u_1(-u_1^2 v_1^2 + a_3 u_1 v_1 - v_1^2 + a_6), \\ v_1'&= Q_1(u_1,v_1)=v_1(v_1^2 - a_3 u_1 v_1 - a_6), \end{aligned} \right. $$

with

$$ D(P_1,Q_1)(0,0) = \begin{bmatrix} a_6 &{} 0 \\ 0 &{} -a_6 \end{bmatrix}. $$

**Fig. 2:**
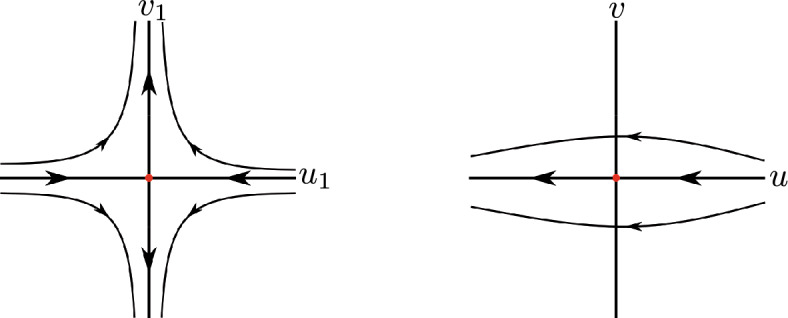
Blow up of the equilibrium point (0, 0) of the local chart $U_1$ in case 1

If $a_6<0$, then $(u_1,v_1)=(0,0)$ is a hyperbolic saddle. Going back through the vertical blow up we obtain that the origin of $U_1$ is formed by two hyperbolic sectors having both separatrices at infinity, see Fig. 2.

In summary, by Proposition 6 system (2) has a global center, and statement (a) of Theorem 4 is proved.

Assume that statement (ii) of Theorem 1 holds. We have $a_6 \ne 0$, otherwise the differential system (2) would be quadratic. System (2) in the chart $U_2$ is

$$ u' = u^2v^2 - a_2 u^2v-a_6u^2+v^2, \qquad v' =uv( v^2-a_2v-a_6). $$

Observe that $v=0$ is the only characteristic direction at the origin of $U_2$. Doing the vertical blow up $(u,v)=(u_1,u_1 v_1)$ and eliminating the factor $u_1$ it follows

$$ u_1' = P_1(u_1,v_1) = u_1(u_1^2 v_1^2 - a_2 u_1 v_1 + v_1^2 - a_6 ), \qquad v_1' = Q_1(u_1,v_1)=-v_1^3, $$

and

$$ D(P_1,Q_1)(0,0) = \begin{bmatrix} -a_6 &{} 0 \\ 0 &{} 0 \end{bmatrix}. $$

If $a_6 > 0$, then $(u_1,v_1)=(0,0)$ is a semi-hyperbolic node, and then system (2) cannot have a global center. If $a_6 < 0$, then $(u_1,v_1)=(0,0)$ is a semi-hyperbolic saddle, and going back through the vertical blow up we conclude that the origin of $U_2$ is formed by two hyperbolic sectors, see Fig. 1.

System (2) in the chart $U_1$ is

$$ u' = -u^2v^2 + a_6 u^2 + a_2 uv - v^2, \qquad v' = -uv^3. $$

Since $u=0$ is not a characteristic direction at the origin of $U_1$, doing the vertical blow up $(u,v)=(u_1,u_1 v_1)$ and eliminating the factor $u_1$ we get the system

$$\begin{aligned}{} & {} u_1' = P_1(u_1,v_1) = u_1(-u_1^2 v_1^2 - v_1^2 + a_2 v_1 + a_6), \\{} & {} \quad v_1' = Q_1(u_1,v_1)=v_1(v_1^2-a_2 v_1-a_6). \end{aligned}$$

This differential system on the straight line $u_1=0$ has the equilibria (0, 0) and $(0,(a_2 \pm \sqrt{a_2^2 + 4 a_6})/2)$ if $a_2^2 + 4 a_6\ge 0$. By Theorem 2.15 of [6] the equilibrium (0, 0) is always a hyperbolic saddle.

When $a_2^2 + 4 a_6> 0$ by Theorem 2.19 of [6] the two equilibria $(0,(a_2 \pm \sqrt{a_2^2 + 4 a_6})/2)$ are semi-hyperbolic saddle-nodes, so the differential system cannot have a global center.

If $a_2^2 + 4 a_6=0$, then doing blow ups the local phase portrait of the equilibrium point $(0,a_2/2)$ is formed by two hyperbolic sectors separatec by two parabolic sectors, so again the differential system cannot have a global center.

Then going back through the vertical blow ups, we conclude that the origin of $U_1$ is formed by two hyperbolic sectors when $a_2^2 + 4 a_6< 0$, see for instance Fig. 2. Therefore statement (c) of Theorem 4 is proved.

If statement (iii) of Theorem 1 holds, then $a_3=a_5=0$ and the study comes down to statement (ii).

Now, suppose that either statement (iv) or (v) of Theorem 1 holds. If $a_2=0$, then $a_5=0$ and consequently the study comes down to statement (i). If $a_2 \ne 0$, then $a_3=a_5=0$ and the study comes down to statement (ii).

*Case* 2. $$a_1^2+4a_4<0$$.

Assume that statement (i) of Theorem 1 holds. We have from (3) that $F(s)= a_4+a_6 s^2$. If $a_6 > 0$ and

$$ p^{\pm } = \pm \sqrt{\dfrac{-a_4}{a_6}}, $$

then $(p^{\pm },0)$ are equilibrium points at infinity in the chart $U_1$ with $F'(p^{\pm }) = 2a_6 p^{\pm } \ne 0$. So from (4) we have that $(p^{\pm },0)$ are semi-hyperbolic saddles, nodes or saddle-nodes. Then system (2) cannot have a global center. So we can suppose that $a_6 \le 0$, and consequently there are no infinite equilibrium points in the chart $U_1$. System (2) in the chart $U_2$ becomes

$$ \begin{aligned} u'&= -a_4u^4 - a_1u^3v+u^2v^2-a_6u^2-a_3uv+v^2,\\ v'&= v(-a_4u^3 - a_1u^2v+uv^2-a_6u-a_3v). \end{aligned} $$

Since $u=0$ is not a characteristic direction at the origin of $U_2$, doing the vertical blow up $(u,v)=(u_1,u_1 v_1)$ and eliminating the factor $u_1$ we obtain

7 $$\begin{aligned} \begin{aligned} u_1'&= P_1(u_1,v_1) = u_1(u^2_1v^2_1 - a_1u^2_1v_1-a_4u_1^2+v^2_1 -a_3v_1-a_6), \\ v_1'&= Q_1(u_1,v_1)=-v_1^3, \end{aligned} \end{aligned}$$

and

$$ D(P_1,Q_1)(0,0) = \begin{bmatrix} -a_6 &{} 0 \\ 0 &{} 0 \end{bmatrix}. $$

If $a_6 < 0$, then $(u_1,v_1)=(0,0)$ is a semi-hyperbolic saddle. Going back through the vertical blow up we conclude that the origin of $U_2$ is formed by two hyperbolic sectors and consequently follows statement (a), see Fig. 1.

Now suppose $a_6=0$. Then both coordinate axes $u_1= 0$ and $v_1=0$ are characteristic directions. Doing the twist $(u_2,v_2)=(u_1+v_1,v_1)$, that translates the direction $u_1=0$ to $u_2=v_2$, system (7) becomes

8 $$\begin{aligned} \begin{array}{rl} u_2' =&{} u^3_2 v^2_2-3u^2_2 v^3_2+3 u_2 v^4_2-v^5_2-a_1 u^3_2 v_2+3a_1 u^2_2 v^2_2-3a_1 u_2 v^3_2+a_1 v^4_2 \\ &{}-a_4u^3_2+3 a_4 u^2_2 v_2+(1-3a_4) u_2 v^2_2+(a_4-2) v^3_2-a_3 u_2 v_2 + a_3 v^2_2, \\ v_2'=&{} -v^3_2. \end{array} \end{aligned}$$

First suppose $a_3 \ne 0$. Then doing the vertical blow up $(u_2,v_2)=(u_3,u_3 v_3)$ in (8) and eliminating the common factor $u_3$ we obtain

9 $$\begin{aligned} \left. \begin{aligned}&\begin{aligned} u_3' =P_3(u_3,v_3)=&u_3[-u^3_3 v^5_3+3 u^3_3 v^4_3 - 3u^3_3 v^3_3 + a_1 u^2_3 v^4_3 + u^3_3 v^2_3 - 3 a_1 u^2_3 v^3_3 \\&+ 3 a_1 u^2_3 v^2_3+(a_4 -2) u_3 v^3_3 - a_1 u^2_3 v_3 + (1 - 3a_4) u_3 v^2_3 \\&+ 3 a_4 u_3 v_3+a_3 v^2_3- a_4 u_3 - a_3 v_3], \end{aligned} \\&\begin{aligned} v_3'=Q_3(u_3,v_3) =&-v_3 (v_3-1)[ -u^3_3 v^4_3 + 2u^3_3 v^3_3 -u^3_3 v^2_3 + a_1 u^2_3 v^3_3 - 2 a_1 u^2_3 v^2_3 \\&+ a_1 u^2_3 v_3 + (a_4-2)u_3 v^2_3 - 2 a_4 u_3 v_3 + a_4 u_3 + a_3 v_3]. \end{aligned} \end{aligned} \right. \end{aligned}$$

So the unique equilibrium points of system (9) in the $v_3-$axis are (0, 0) and (0, 1). Since

$$ D(P_3,Q_3)(0,1) = \begin{bmatrix} 0 &{} 0 \\ 0 &{} -a_3 \end{bmatrix} $$

the equilibrium (0, 1) is a semi-hyperbolic saddle-node. Consequently system (2) cannot have a global center.

Now assume that $a_3=0$. Then we can eliminate another common factor $u_3$ in system (9) and we have

10 $$\begin{aligned} \left. \begin{aligned}&\begin{aligned} u_3' =P_3(u_3,v_3)=&u_3[-u^2_3 v^5_3+3 u^2_3 v^4_3 - 3u^2_3 v^3_3 + a_1 u_3 v^4_3 + u^2_3 v^2_3 - 3 a_1 u_3 v^3_3 \\&+ 3 a_1 u_3 v^2_3+(a_4 -2) v^3_3 - a_1 u_3 v_3 + (1 - 3a_4) v^2_3 \\&+ 3 a_4 v_3- a_4], \end{aligned} \\&\begin{aligned} v_3'=Q_3(u_3,v_3) =&-v_3 (v_3-1)[ -u^2_3 v^4_3 + 2u^2_3 v^3_3 -u^2_3 v^2_3 + a_1 u_3 v^3_3 - 2 a_1 u_3 v^2_3 \\&+ a_1 u_3 v_3 + (a_4-2) v^2_3 - 2 a_4 v_3 + a_4]. \end{aligned} \end{aligned} \right. \end{aligned}$$

Then the equilibrium points $(0,v_3)$ of system (10) are determined by the zeros of the polynomial

$$ v_3 (v_3-1)p(v_3)=0, $$

where $p(v_3)=(a_4-2) v^2_3 - 2a_4 v_3+a_4$. Since $a_4 < 0$ and the discriminant of *p* is $8 a_4$ it follows that (0, 0) and (0, 1) are the unique equilibrium points of system (10), with

$$ D(P_3,Q_3)(0,0) = \begin{bmatrix} -a_4 &{} 0 \\ 0 &{} a_4 \end{bmatrix} \text{ and } D(P_3,Q_3)(0,1) = \begin{bmatrix} -1 &{} 0 \\ 0 &{} 2 \end{bmatrix}. $$

**Fig. 3:**
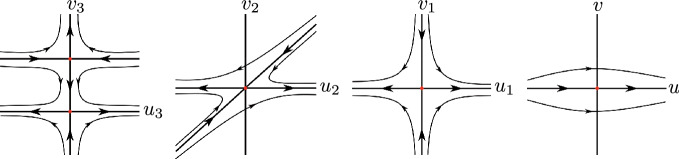
Blow up’s of the equilibrium point (0, 0) of the local chart $U_2$ under the case 2 with $a_3=a_6=0$

Therefore both equilibrium points are hyperbolic saddles. Going back through the changes of variables we obtain that the origin of the chart $U_2$ is formed by two hyperbolic sectors. Therefore statement (b) is proved (Fig. 3).

Suppose (ii) holds in Theorem 1. System (2) in the chart $U_2$ writes

$$ u' = -a_4u^4 +u^2v^2- a_2 u^2 v -a_6 u^2 + v^2, \quad v' = uv(-a_4u^2 +v^2-a_2v-a_6). $$

Now, doing the vertical blow up $(u,v)=(u_1,u_1 v_1)$ and eliminating the common factor $u_1$ we get

$$ u_1' = P_1(u_1,v_1) = u_1(u^2_1 v^2_1 -a_4 u^2_1 - a_2 u_1 v_1 +v^2_1 - a_6), \quad v_1' = Q_1(u_1,v_1)=-v_1^3, $$

and

$$ D(P_1,Q_1)(0,0) = \begin{bmatrix} -a_6 &{} 0 \\ 0 &{} 0 \end{bmatrix}. $$

Therefore if $a_6 > 0$, then $(u_1,v_1)=(0,0)$ is a semi-hyperbolic node, and consequently the center of system (2) cannot be global. So, since $a_4<0$ and $a_6\le 0$, this implies that there are no infinite equilibrium points on the local chart $U_1$.

If $a_6 < 0$, then $(u_1,v_1)=(0,0)$ is a semi-hyperbolic saddle and going back through the vertical blow up we have that the origin of $U_2$ is formed by two hyperbolic sectors, see Fig. 1. Then statement (d) follows.

Assume $a_6=0$. Doing the change of variables $(u_2,v_2)=(u_1+v_1,v_1)$ and then doing the vertical blow up $(u_2,v_2)=(u_3,u_3 v_3)$ and eliminating the commun factor $u^2_3$ we obtain

11 $$\begin{aligned} \left. \begin{aligned}&\begin{aligned} u_3' =P_3(u_3,v_3)=&-u_3[u^2_3 v^5_3-3 u^2_3 v^4_3 + 3 u^2_3 v^3_3- u^2_3 v^2_3 + (a_2 - a_4 +2)v^3_3 \\&+(3 a_4 - 2 a_2 -1)v^2_3 + (a_2 - 3 a_4)v_3 + a_4], \end{aligned} \\&\begin{aligned} v_3'=Q_3(u_3,v_3) =&v_3 (v_3-1)[ u^2_3 v^4_3 - 2u^2_3 v^3_3 + u^2_3 v^2_3+ (a_2 - a_4 +2)v^2_3 \\&+(2 a_4 - a_2)v_3 - a_4]. \end{aligned} \end{aligned} \right. \end{aligned}$$

The equilibrium points of system (11) are (0, 0), (0, 1) and the points $(0,v_3)$ such that $v_3$ be a real zero of the polynomial

$$ q(v_3)= (a_2-a_4+2) v^2_3 + (2 a_4 -a_2)v_3-a_4=0. $$

The points (0, 0) and (0, 1) are hyperbolic saddles because

$$ D(P_3,Q_3)(0,0) = \begin{bmatrix} -a_4 &{} 0 \\ 0 &{} a_4 \end{bmatrix} \text{ and } D(P_3,Q_3)(0,1) = \begin{bmatrix} -1 &{} 0 \\ 0 &{} 2 \end{bmatrix}. $$

First we assume that $a_2=a_4-2$. Suppose $a_4 \ne -2$. Then

$$ p_0=\left( 0,\dfrac{a_4}{a_4 + 2}\right) $$

is an equilibrium point of system (11) with

$$ D(P_3,Q_3)(p_0) = \begin{bmatrix} -\dfrac{a^2_4}{(a_4 + 2)^2} &{} 0 \\ 0 &{} -\dfrac{2a_4}{a_4 + 2} \end{bmatrix}. $$

**Fig. 4:**
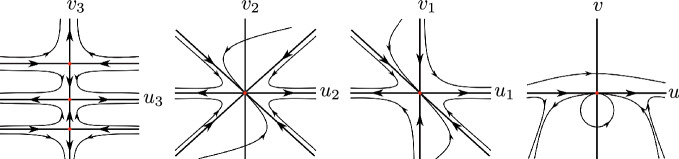
Blow-up’s of the equilibrium point (0, 0) of the local chart $U_2$ under the case 2 with $a_2=a_4-2$ and $-2<a_4<0$

If $a_4<-2$, then $p_0$ is a hyperbolic stable node and system (2) cannot have a global center. If $-2<a_4<0$, then $p_0$ is a hyperbolic saddle. However, going back through the change of variables there are trajectories that tend to the origin of $U_2$, see Fig. 4. Hence the center of system (2) cannot be global.

When $a_4=-2$, the unique infinite equilibrium points of system (11) are (0, 0) and (0, 1). Going back through the changes of variables it follows that the origin of $U_2$ has an elliptic sector, so the differential system cannot have a global center, see Fig. 4.

Suppose $a_4 \ne a_2+2$. The discriminant of the polynomial *q* is $a^2_2 + 8 a_4$.

Assume $a^2_2 + 8 a_4 =0$. Then, since $a_4<0$ we have that $a_2 \ne -4,0$, and

$$ q_0=\left( 0,\dfrac{a_2}{a_2+4}\right) $$

is an equilibrium point of system (11) with

$$ D(P_3,Q_3)(q_0) = \begin{bmatrix} -\dfrac{a^2_2}{(a_2 + 4)^2} &{} 0 \\ 0 &{} 0 \end{bmatrix}. $$

Note that the vector field over the line $u_3=0$ is given by

$$ (P_3(0,v_3),Q_3(0,v_3)) = \left( 0, \dfrac{1}{8} v_3 (v_3-1) [(a_2+4) v_3 - a_2]^2\right) . $$

Consequently, $q_0$ is a semi-hyperbolic saddle-node and system (2) cannot have a global center, because going back through the changes of variables there are orbits which go or come from the origin of the local chart $U_2$.

Now assume that $a^2_2 + 8 a_4 >0$. So $a_2\ne 0$ because $a_4<0$. Then

$$ p^{\pm }=\left( 0, \dfrac{a_2 - 2a_4 \pm \sqrt{a_2^2+8 a_4}}{2(a_2-a_4+2)} \right) $$

are equilibrium points of system (11). Denote $a_2^2+8 a_4=b^2$ with $b>0$. Then

$$ D(P_3,Q_3)(p^{-}) = \begin{bmatrix} -\dfrac{(a_2 - b)^2 (a_2 + b + 4)^2}{32} &{} 0 \\ 0 &{} \dfrac{b(a_2 - b) (a_2 + b + 4)^2}{8} \end{bmatrix}, $$

where $$-(a_2 - b)^2 (a_2 + b + 4)^2/32<0$$.

If $a_2 < 0$ it follows that $b(a_2 - b) (a_2 + b + 4)^2/8 < 0$, and then $p^{-}$ is a hyperbolic stable node and system (2) cannot have a global center.

If $a_2>0$, then we have

$$ D(P_3,Q_3)(p^{+}) = \begin{bmatrix} -\dfrac{(a_2 + b)^2 (a_2 - b + 4)^2}{32} &{} 0 \\ 0 &{} -\dfrac{b(a_2 + b) (a_2 - b + 4)^2}{8} \end{bmatrix}, $$

with $-(a_2 + b)^2 (a_2 - b + 4)^2/32 < 0$ and $-b(a_2 + b) (a_2 - b + 4)^2/8 < 0$. Thus $p^{+}$ is a hyperbolic stable node and, consequently system (2) cannot have a global center.

Finally assume that $a^2_2 + 8 a_4 <0$. Then (0, 0) and (0, 1) are the unique equilibrium points, because the polynomial *q* is positive. Going back through the changes of variables we obtain that the origin of $U_2$ is formed by two hyperbolic sectors. Therefore this completes the proof of statement (e).

Observe that statement (iii) of Theorem 1 cannot hold in *Case* 2, otherwise $a^2_1 + 4 a_4 = (a_1 + 2 a_3)^2 \ge 0$, in contradiction with the fact that $a^2_1 + 4 a_4<0$.

Assume that statement (iv) holds in Theorem 1. If $a_2 \ne 0$ we obtain

$$ a_6 = -\dfrac{2 a^2_2 + 9 a^2_3}{9},\quad a_4=a_3 (a_1 + a_3) \ \ \text{ and } \ \ a_1 = -\dfrac{a_3 (9 a^2_3 + 4 a^2_2)}{2 a^2_2}. $$

Therefore $$81 a^6_3/(4 a^4_2)=a^2_1 + 4 a_4 < 0$$, a contradiction.

Assume now that $a_2=0$. Then the study boils down to studying global centers under condition (i).

Suppose that statement (v) in Theorem 1 holds. Assume $$a_2 \ne 0$$. Then $$a_1=-a_3$$, $$a_5=a_6=0$$, $$a_4=-(2a^2_2 + 9 a^2_3)/18$$ and $$ a_3(2 a^2_2 + 9 a^2_3)/2=0$$. Hence $$a_3=0$$ and the only infinite equilibrium point is the origin of the chart $$U_2$$. System (2) in the chart $$U_2$$ is

$$ u' = \dfrac{a^2_2 u^4}{9} +u^2 v^2- a_2 u^2 v + v^2, \quad v' = \dfrac{uv(a^2_2 u^2 + 9 v^2 - 9 a_2 v)}{9}. $$

Doing the vertical blow up $(u,v) = (u_1,u_1 v_1)$ and eliminating the common factor $u_1$ between $u_1'$ and $v_1'$ we get

$$ u_1' = P_1(u_1,v_1) = \dfrac{u_1(9 u^2_1 v^2_1 + a^2_2 u^2_1 -9 a_2 u_1 v_1 + 9 v^2_1}{9}, v_1' = Q_1(u_1,v_1)=-v_1^3. $$

Doing the change of variables $(u_2,v_2)=(u_1+v_1,v_1)$, after doing the vertical blow up $(u_2,v_2)=(u_3,u_3 v_3)$, and eliminating the commun factor $u^2_3$, we obtain

12 $$\begin{aligned} \begin{aligned}&\begin{aligned} u_3' =P_3(u_3,v_3)=&-\dfrac{u_3}{9}[ 9 u^2_3 v^5_3 - 27 u^2_3 v^4_3 + 27 u^2_3 v^3_3-9 u^2_3 v^2_3 \\&+ (a^2_2+ 9a_2 + 18) v^3_3 - (3 a^2_2 + 18 a_2 + 9) v^2_3 \\&+ (3 a^2_2 + 9 a_2) v_3-a^2_2], \end{aligned} \\&\begin{aligned} v_3'=Q_3(u_3,v_3) =&\dfrac{v_3(v_3-1)}{9}[ 9 u^2_3 v^4_3 - 18 u^2_3 v^3_3 + 9 u^2_3 v^2_3 \\&+ (a^2_2 + 9a_2 + 18) v^2_3- (2 a^2_2 + 9 a_2)v_3 + a^2_2]. \end{aligned} \end{aligned} \end{aligned}$$

Thus the equilibrium points of system (12) are (0, 0), (0, 1) and the points $(0,v_3)$ such that $v_3$ is a real zero of the polynomial

$$ (a_2+6)(a_2+3) v^2_3 - a_2 (2 a_2 + 9) v_3 + a^2_2=0. $$

We have

$$ D(P_3,Q_3)(0,0) = \begin{bmatrix} \dfrac{a^2_2}{9} &{} 0 \\ 0 &{} -\dfrac{a^2_2}{9} \end{bmatrix} \text{ and } D(P_3,Q_3)(0,1) = \begin{bmatrix} -1 &{} 0 \\ 0 &{} 2 \end{bmatrix}, $$

i.e., (0, 0) and (0, 1) are hyperbolic saddles.

If $a_2=-6$, then (0, 2) is a hyperbolic stable node, and consequently system (2) cannot have a global center.

If $a_2=-3$, then $(0,-1)$ is a hyperbolic saddle with

$$ D(P_3,Q_3)(0,-1) = \begin{bmatrix} -1 &{} 0 \\ 0 &{} 2 \end{bmatrix}. $$

Going back through the changes of variables we obtain that there are trajectories that tend to the origin of $U_2$, so system (2) cannot have a global center.

Now if $(a_2+6)(a_2+3) \ne 0$, then the point $(0,a_2/(a_2+3))$ is a hyperbolic stable node and system (2) cannot have a global center.

Finally, if $a_2=0$ then $a_5=0$ the study boils down to studying global centers under statement (i) of Theorem 1. $\square$
